# New improved gamma: Enhancing the accuracy of Goodman–Kruskal’s gamma using ROC curves

**DOI:** 10.3758/s13428-018-1125-5

**Published:** 2018-09-27

**Authors:** Philip A. Higham, D. Paul Higham

**Affiliations:** 1 Department of Psychology, University of Southampton, Southampton, UK; 2 Cupertino, CA USA

**Keywords:** Resolution, Gamma, ROC curves, Trapezoidal rule, Metacognition

## Abstract

For decades, researchers have debated the relative merits of different measures of people’s ability to discriminate the correctness of their own responses (resolution). The probabilistic approach, primarily led by Nelson, has advocated the Goodman–Kruskal gamma coefficient, an ordinal measure of association. The signal detection approach has advocated parametric measures of distance between the evidence distributions or the area under the receiver operating characteristic (ROC) curve. Here we provide mathematical proof that the indices associated with the two approaches are far more similar than has previously been thought: The true value of gamma is equal to twice the true area under the ROC curve minus one. Using this insight, we report 36 simulations involving 3,600,000 virtual participants that pitted gamma estimated with the original concordance/discordance formula against gamma estimated via ROC curves and the trapezoidal rule. In all but five of our simulations—which systematically varied resolution, the number of points on the metacognitive scale, and response bias—the ROC-based gamma estimate deviated less from the true value of gamma than did the traditional estimate. Consequently, we recommend using ROC curves to estimate gamma in the future.

**Electronic supplementary material:**

The online version of this article (10.3758/s13428-018-1125-5) contains supplementary material, which is available to authorized users.

An important question in many domains of psychology is whether people are metacognitively accurate. One type of metacognitive accuracy is *resolution*, which is the degree to which a metacognitive rating discriminates between a person’s own correct versus incorrect responses. For example, people may rate how confident they are in a particular response on a 1 to 6 scale (6 = *highest confidence*). If, on average, accurate responses are assigned higher values on the scale than inaccurate ones, then resolution is good. Resolution is best if people use the extremes of the scale to discriminate correctness. For example, someone who assigns “6” to all her accurate responses and “1” to all her inaccurate ones is demonstrating perfect resolution. The same principle applies to other metacognitive ratings, such as judgments of learning (JOLs) and feelings of knowing.

Resolution is considered important because it affects *control* (Nelson & Narens, 1990). For example, students writing a multiple-choice test for which errors are penalized but omissions are not face a metacognitive decision: Is the candidate answer under consideration for a question accurate or not (e.g., Higham, 2007)? If it is assessed as correct, students may well risk the penalty and offer it as a response. However, if it is assessed as incorrect, the decision may be to withhold the response. Clearly, resolution determines whether the decision to report (or withhold) the answer increases the test score. A student with perfect resolution will offer all her correct responses and withhold all her incorrect ones, resulting in the highest score possible given her knowledge. Conversely, another student with equal knowledge may score lower on the test if her resolution is poor. With poor resolution, the student may offer a portion of her incorrect candidate responses and withhold some of her correct ones, resulting in penalties and lost opportunities for points, respectively (see Arnold, Higham, & Martín-Luengo, 2013; Higham, 2007; Higham & Arnold, 2007, for discussion of the metacognitive processes involved in formula-scored tests).

Given the importance of resolution for understanding metacognitive processes and people’s behavior, it is critical that it be measured properly. However, the best index of resolution has been an issue of ongoing debate (e.g., Higham, 2007, 2011; Higham, Zawadzka, & Hanczakowski, 2016; Masson & Rotello, 2009; Nelson, 1984, 1986, 1987; Rotello, Masson, & Verde, 2008; Swets, 1986). On the one hand, there are proponents of Goodman–Kruskal’s gamma coefficient (Goodman & Kruskal, 1954), an ordinal measure of association ranging between – 1 (*perfect negative relationship*) and + 1 (*perfect positive relationship*). One such highly influential proponent was Nelson (1984), who compared a variety of different measures of association and advocated gamma for a number of reasons. First, it made no scaling assumptions beyond the data being ordinal. Second, it could achieve its highest value possible (+ 1) under most circumstances. Third, it could be computed from data arranged in a number of different table formats (e.g., 2 × 2 tables or 2 × *R* tables, where *R* > 2). By far, this index continues to be the most common measure of resolution in the metacognitive literature. Nelson’s (1984) review of potential measures of resolution and ultimate promotion of gamma as the best one has had tremendous impact on the field since it was first published.

On the other hand, other researchers and statisticians have recommended signal detection theory (SDT) as an alternative to gamma (e.g., Benjamin & Diaz, 2008; Higham, 2011; Higham et al., 2016; Masson & Rotello, 2009; Rotello et al., 2008; Swets, 1986). Resolution is a discrimination task—people must discriminate the correctness of their own responses—so a suitable measure based on SDT seems like an obvious choice, given that this theory was designed to provide a pure measure of discrimination, free from response bias. Proponents of SDT have argued that, unlike SDT measures such as *A*_*z*_ or *d*_*a*_, gamma is contaminated by response bias (e.g., Masson & Rotello, 2009). However, despite clear demonstrations of this fact, as well as other undesirable properties such as a tendency to produce Type I inferential errors (Rotello et al., 2008), gamma continues to be used pervasively throughout the metacognitive literature.

The purpose of the present article is to contribute to this debate regarding the best measure of resolution in a unique way; we highlight similarities rather than differences between the measures. By sidestepping the typically confrontational nature of this debate (see, in particular, the exchanges between Nelson and Swets in the 1980s; e.g., Nelson, 1986, 1987; Swets, 1986), we hope to encourage new insights not only regarding which measure of resolution is the best one to use in a given situation, but also to demonstrate how it is possible to translate one measure from the so-called *probabilistic* approach involving gamma to SDT measures, and vice versa. By emphasizing the similarities between the measures rather than their differences, we introduce a new computational formula for gamma that is based on SDT. Our simulations show that when this SDT-based formula is used instead of the one suggested by Goodman and Kruskal (1954), which is derived from concordant and discordant pairs of observations (explained next), the estimates of gamma obtained from sample data deviate far less from the true value.

## Traditional gamma: Concordant and discordant pairs of observations

In this section, we briefly review the original computational formula for gamma introduced by Goodman and Kruskal (1954), and its limitations. Suppose that experimental participants are presented with a list of 50 unrelated cue–target pairs, such as *digit–hungry*. Following presentation of each pair, participants are asked to judge the likelihood (using a 0%–100% scale) that they will recall the target if presented with the cue in a cued-recall memory test held at the end of the experiment—a so-called *judgment of learning* (JOL). On the cued-recall test, suppose that one participant recalled 30 of the targets from the 50 cues on the test (60% accuracy). The participant’s 30 correct and 20 incorrect recall attempts can then be tabulated contingent on the JOLs she made during study. Suppose that the JOLs, which can assume any integer value between 0 and 100, are divided into ten bins, as in Table 1. Binning data in this way is a common procedure in metacognitive research, used to, for example, construct calibration curves. To compute gamma using the original formula, one first determines the total number of *concordant* (C) and *discordant* (D) pairs of observations. These terms refer to the ordering of the two observations within the pair on the two variables. If the ordering of the two observations on one variable is the same as the ordering on the second variable, then there is a concordance (e.g., JOL_a_ > JOL_b_ and Recall_a_ > Recall_b_, where a and b refer to items within the pair). Alternatively, if the ordering of the two observations on the two variables are opposite (e.g., JOL_a_ > JOL_b_ and Recall_a_ < Recall_b_), then there is a discordance. In Table 1, the concordant pairs would be those for which the JOL assigned to a correct response exceeds that assigned to an incorrect response. Discordant pairs, on the other hand, are those for which the JOL assigned to an incorrect response exceeds that assigned to a correct response. The numbers of concordant and discordant pairs for the data in Table 1 are shown at the bottom of the table. Gamma is then computed as the number of concordant pairs minus the number of discordant pairs, all divided by the total number of concordant and discordant pairs—that is,

1 $$ \mathrm{Gamma}=\frac{C-D}{C+D} $$

**Table 1:** Hypothetical frequency table showing the number of correctly recalled and not recalled responses distributed across ten JOL bins

	JOL Bin										
Accuracy	0–9	10–19	20–29	30–39	40–49	50–59	60–69	70–79	80–89	90–100	Total
Recalled (correct)	0	0	1	2	2	4	3	3	4	11	30
Not recalled (incorrect)	7	5	2	2	1	1	2	0	0	0	20

The numbers of concordant and discordant pairs computed, along with gamma estimated using the original formula: Concordant pairs = C = 7(0+1+2+2+4+3+3+4+11) + 5(1+2+2+4+3+3+4+11) . . . 0(11) = 554; Discordant pairs = D = 5(0) + 2(0+0) + 2(0+0+1) . . . 0(0+0+1+2+2+4+3+3+4) = 28; Ties = T = 0.5*N*(*N* – 1) – C – D = 1,225 – 554 – 28 = 643, where *N* equals the total number of observations; Gamma = (C – D)/(C + D) = (554 – 28)/(554 + 28) = .904; JOL = judgment of learning

In the example shown in Table 1, gamma is equal to .904. This value corresponds to excellent resolution, since the maximum value that gamma can assume is 1.0. This excellent resolution can be intuited by noting that the JOLs for correct versus incorrect responses tend to be clustered toward the top versus the bottom of the scale, respectively. In other words, correct responses tend to be assigned high JOLs, whereas incorrect responses tend to be assigned low JOLs, showing that this participant was metacognitively accurate in predicting her future memory performance.

Now consider the same participant’s data divided into five bins instead of ten, a scenario depicted in Table 2. One might expect that the gamma computed from the data in Table 2 would also be .904 as it was in Table 1, given that the two tables are based on exactly the same data; the only difference between the tables is the seemingly arbitrary decision about how to bin the data. However, reducing the number of bins reduces both the number of concordant pairs (540 instead of 554) and the number of discordant pairs (24 instead of 28). This has the effect of increasing gamma from .904 to .915. At the extreme, where there are only two bins corresponding to, say, JOL < 50 and JOL ≥ 50, producing a 2 × 2 table, there would be only 425 concordant pairs and 15 discordant pairs, to yield gamma = .932. In short, the fewer the bins for a given data set, the greater the distortion of gamma if it is computed with the original formula.

**Table 2:** Hypothetical frequency table showing the number of correctly recalled and not recalled responses distributed across five JOL bins

	JOL Bin					
Accuracy	0–19	20–39	40–59	60–79	80–100	Total
Recalled (correct)	0	3	6	6	15	30
Not recalled (incorrect)	12	4	2	2	0	20

These are the same data as in Table 1, except there are fewer bins. The numbers of concordant and discordant pairs computed, along with gamma estimated using the traditional formula: Concordant pairs = C = 12(3+6+6+15) + 4(6+6+15) . . . 2(15) = 540; Discordant pairs = D = 4(0) + 2(0+3) + 2(0+3+6) . . . 0(0+3+6+6) = 24; Ties = T= 0.5*N*(*N* – 1) – C – D = 1225 – 540 – 24 = 661, where *N* equals the total number of observations; Gamma = (C – D)/(C + D) = (540 – 24)/(540 + 24) = .915; JOL = judgment of learning

The reason why reducing the number of confidence bins distorts gamma is that it increases the total number of *ties* (T)—that is, pairs of observations that do not differ on one, the other, or both the JOL and recall accuracy variables. Referring to the tables again, some pairs that were either concordant or discordant in Table 1 are tied in Table 2. The number of ties can be computed by subtracting the numbers of concordant and discordant pairs from the total number of pairs (i.e., T = 0.5*N*[*N* – 1] – C – D, where *N* equals the total number of observations). Out of the 1,225 total pairs in the data set used to generate Tables 1 and 2 (50[49]/2 = 1,225), there are 643 ties in Table 1 with ten bins, 661 in Table 2 with five bins, and 785 in the 2 × 2 case (if confidence is split at 50%). There are three types of ties (Gonzalez & Nelson, 1996): pairs that are tied on (1) the metacognitive judgment (i.e., the two JOLs are in the same bin) but not the recall test (i.e., one is correct, but the other is not); (2) the recall test (i.e., both correct or both incorrect) but not the metacognitive judgment (i.e., the two JOLs are in different bins); and (3) both variables (i.e., pairs assigned the same JOL, which are both correct or both incorrect). In Table 2, the 661 total ties are made up of 212, 36, and 413 ties of these three types, respectively. However, regardless of the particular nature of the ties caused by decreasing the number of bins, the effect on gamma is the same: Ties mean that gamma is distorted. Only in the case of no ties is the value of gamma accurate (Masson & Rotello, 2009).

The problem of tied observations and their effect on gamma has been known for some time. Potential solutions have been offered that typically entail including some of the tied pairs in the denominator of the computational formula for gamma, thereby reducing the overestimation (e.g., Kim, 1971; Somers, 1962; Wilson, 1974; see Freeman, 1986, for a review). The purpose of our commentary is not to adjudicate on which correction might be the most suitable. Rather, we wish to offer an alternative method for computing gamma that does not involve ties; indeed, it does not involve the notion of concordant and discordant pairs at all, and is therefore free of the problems inherent in the original formula.

## V: The proportion of concordant pairs

Nelson (1984) described a statistic that is closely related to gamma: *V*, the proportion of concordant pairs. In an ideal circumstance in which there are no ties, then

2 $$ V=\frac{C}{C+D} $$

If there are no ties, the proportion of concordant pairs (*V*) and the proportion of discordant pairs are complementary, such that

3 $$ \left(1-V\right)=\frac{D}{C+D} $$

Nelson showed that, because gamma is equal to Eq. 2 minus Eq. 3 (i.e., the difference in the proportions of concordant and discordant pairs),

4 $$ \mathrm{Gamma}=V-\left(1-V\right)=2V-1 $$

The relevance of *V* and Eq. 4 will become apparent later.

## Alternatives to gamma: Signal detection theory

Adopting a signal detection framework, Masson and Rotello (2009) showed that gamma is contaminated by response bias. In the metacognitive context, liberal versus conservative response biases would be represented in Table 1 as a clustering of observations in the bins associated with high versus low confidence values, respectively. At the extreme, maximally liberal versus maximally conservative responding would result in all observations falling into the 90–100 bin versus the 0–10 bin, respectively. At these extremes, *all* the observations are ties, with the number of ties reducing as the clustering is reduced. As an alternative to gamma, Masson and Rotello recommended parametric signal detection measures such as *d*_*a*_ or *A*_*z*_, which are free of response bias if the parametric assumptions are met. However, as we discuss in more detail later, these measures present their own practical as well as potential theoretical problems.

We now turn to the area under the receiver operating characteristic (ROC) curve, which *A*_*z*_ estimates. ROC curves, introduced to psychology from engineering in the 1950s, are now used widely in both experimental psychology and medicine, as they provide a great deal of useful information about discrimination performance. In short, an ROC curve is a plot of the hit rate (HR) as a function of the false alarm rate (FAR) at different levels of response bias. Within the metacognitive context, the HR and the FAR are the conditional probabilities that participants identified correct and incorrect responses, respectively, as correct. There are a variety of ways that a response might be identified as correct. Participants may choose to report (rather than withhold) an answer in a formula-scored testing situation, or they may respond “yes” when asked if they are confident in their answer. However, identification of correct answers using binary responses (report/withhold or yes/no), by itself, only produces one point for the ROC curve, because it produces only one HR and FAR pair. To generate several points for the ROC, which gives a better indication of its shape, confidence ratings are commonly used.

To illustrate a confidence-based ROC curve, consider again the data in Table 1. The first step in creating an ROC curve of these data is to generate a table of the cumulative frequencies, shown in panel A of Table 3. Starting at the highest level of confidence and moving to lower confidence levels, observations are accumulated until all of the observations are represented at the lowest confidence level. The cumulative nature of the data in Table 3 is indicated by the “+” sign following each confidence level. For example, the column corresponding to “70+” includes all the correct and incorrect responses assigned a confidence level of 70 or higher. For the column “0+,” all responses are assigned a confidence level of 0 or higher; hence, the values in that column match the row totals at the right-hand end of the row.

**Table 3:** Hypothetical cumulative frequency table (panel A), showing the numbers of correctly recalled and not recalled responses that are equal to or higher than the designated confidence criteria, along with the hit rates and false alarm rates (panel B) derived from the cumulative frequencies in panel A

	Confidence Criteria										
	0+	10+	20+	30+	40+	50+	60+	70+	80+	90+	Total
A – Cumulative Frequencies											
Recalled (correct)	30	30	30	29	27	25	21	18	15	11	30
Not recalled (incorrect)	20	13	8	6	4	3	2	0	0	0	20
B – Rates											
Hit rates	1.00	1.00	1.00	.97	.90	.83	.70	.60	.50	.37	–
False alarm rates	1.00	.65	.40	.30	.20	.15	.10	.00	.00	.00	–

These values are based on the noncumulative data shown in Table 1. The plus signs next to the confidence criteria in each panel indicate that the data are cumulative.

Next, the cumulative frequencies are converted into rates, shown in panel B of Table 3. Specifically, the cumulative frequencies are divided by the total number of observations of a given type. Correct responses yield HRs, whereas incorrect responses yield FARs. Note that the rates for higher confidence levels generally are smaller than those at lower confidence levels. This mapping corresponds to more conservative responding versus more liberal responding, respectively. A way to understand the table of HRs and FARs is to treat decreasing levels of confidence as decreasing levels of conservatism. That is, for confidence level “90+,” it is as if participants are only identifying as correct those responses assigned 90 or higher. On the other hand, for confidence level “30+,” it is as if participants are identifying as correct those responses assigned 30 or higher, which means that more items have been identified as correct (for 90+ vs. 30+, respectively: HRs, .37 vs. .97; FARs, 0 vs. .30).

**Fig. 1:**
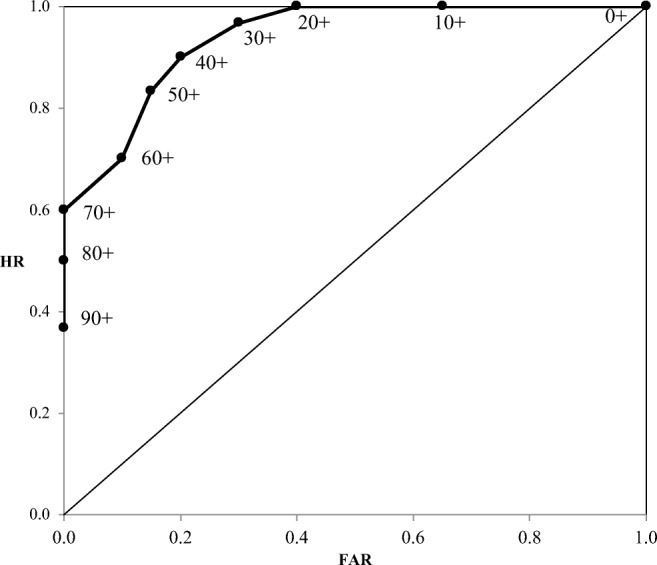
Hypothetical receiver operating characteristic (ROC) curve based on the hit rates and false alarm rates shown in panel B of Table 3. The plus signs next to the points on the ROC indicate that the rates are cumulative. HR = hit rate, FAR = false alarm rate

The values in the rates table can then be plotted in a unit space, with FARs on the *x*-axis and HRs on the *y*-axis. The ROC curve for the data shown in panel B of Table 3 is shown in Fig. 1. A number of interesting performance metrics can be gleaned from the ROC curve. Note that if participants were completely unable to discriminate between their own correct and incorrect responses, the HR and FAR would be equal to each other. In other words, correct responses would be just as likely to be identified as correct as incorrect. By convention, chance performance is depicted in the ROC space as the diagonal line, commonly referred to as the *chance diagonal*. Note, however, that the actual ROC curve is bowed away from the chance line. This bowing indicates that discrimination is above chance, because the HRs exceed the FARs at all confidence levels. Because more bowing is indicative of better discrimination, area under the curve (AUC) provides a useful measure of discrimination. *A*_*z*_, mentioned earlier, is a measure of this area and can be obtained from sample data using maximum-likelihood estimation if it is assumed that there are Gaussian correct and incorrect response distributions. Such an assumption may not be valid in the context of metacognitive discrimination (resolution), a point to which we will return later. A nonparametric alternative is *A*_*g*_, which estimates the area by connecting the points on the ROC curve (as well as the [0,0] point) with straight lines and computing the area using the trapezoidal rule (Pollack & Hsieh, 1969). In particular, the formula for *A*_*g*_ is

5 $$ {A}_g=0.5\sum \limits_{k=0}^n\left({HR}_{k+1}+{HR}_k\right)\left({FAR}_{k+1}-{FAR}_k\right), $$

where *k* represents the different criteria plotted on the ROC and *n* is the number of criteria. Therefore, for the ROC curve in Fig. 1, which is based on the data in Table 3,

6 $$ {A}_g=\left(1+1\right)\left(1-.65\right)+\left(1+1\right)\left(.65-.40\right)\cdots +\left(.37+0\right)\left(0-0\right)=.94 $$

## The relationship between V and area under the ROC curve

**Fig. 2:**
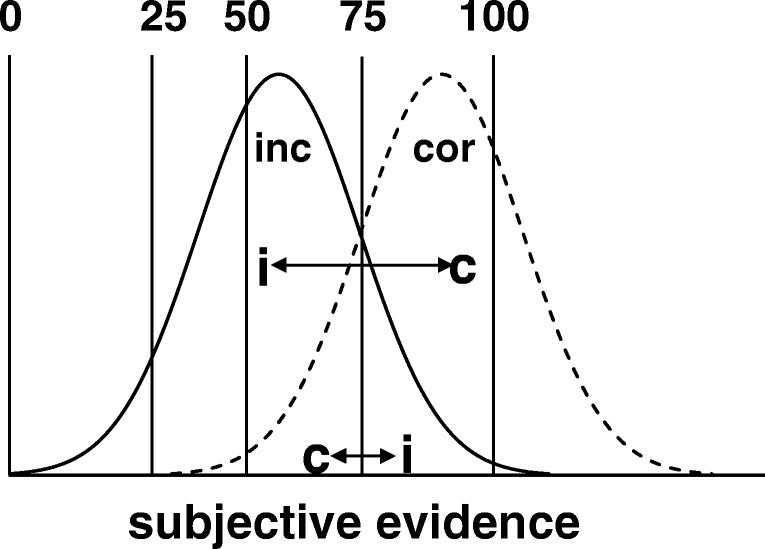
Signal detection model showing incorrect (inc) and correct (cor) items distributed normally over the subjective evidence (for correctness). The vertical lines represent the confidence criteria associated with confidence levels 0, 25, 50, 75, and 100. The *c* and *i* pairs joined by the horizontal, double-headed arrows represent pairs of observations drawn at random, one each from the correct and incorrect item distributions, respectively. In the upper case, *c* has more evidence than *i*, making the pair concordant. However, the opposite is true in the bottom case, making the pair discordant

Figure 2 shows another way to depict monitoring and confidence in a signal detection model. The model assumes that there is an underlying dimension constituting the subjective evidence (for correctness).^1^ In most cases, correct items (in the current context, those that are successfully recalled) have more subjective evidence than incorrect items. The vertical lines represent different confidence criteria. Thus, for an item to be assigned 75%, it must be associated with enough evidence to equal or exceed the 75% confidence criterion, but not to equal or exceed the 100% confidence criterion (in which case it would be assigned 100%). Note that there are only five criteria in this example, rather than ten as in Fig. 1 and Table 3. The number of criteria was reduced simply to avoid the figure seeming too busy and is not important for the present purposes.

One interpretation of the area under the ROC curve (which *A*_*g*_ estimates) is that it is equal to the likelihood that an observation drawn at random from the correct item distribution will be higher on the subjective evidence dimension than an observation drawn at random from the incorrect item distribution. In Fig. 2, two such pairs are shown, as *c* (a correct item drawn at random) and *i* (an incorrect item drawn at random), joined by a line with two arrow heads to indicate that they are part of the same pair. In the upper example, *c* exceeds *i*. In bottom example, the opposite is true. It is straightforward to see that as the distributions separate, such that there is less overlap, cases of *c* > *i* will increasingly prevail over cases of *c* < *i* , until *P*(*c* > *i*) = 1. In other words, with no overlap of the distributions, there is perfect discrimination, and AUC will also be equal to 1. Conversely, if the distributions are drawn together until they completely overlap, then *P*(*c* > *i*) = *P*(*c* < *i*) = .5, which is also equal to AUC (chance diagonal). We provide a mathematical proof that *P*(*c* > *i*) is equal to AUC in the supplementary materials.

There is another way to interpret these pairs of observations and how they compare on the subjective evidence dimension. Specifically, for the *c* > *i* pairs, both confidence and accuracy are higher for *c* than for *i*, making the observation pair concordant. In contrast, for the *c* < *i* pairs, *c* is less than *i* on confidence, but higher than *i* on accuracy, making the pair discordant. Equation 2 indicates that the proportion of concordant pairs in the entire sample (i.e., *P*[*c* > *i*]) is equal to *V*. However, above we noted that *P*(*c* > *i*) is equal to AUC. Thus,

7 $$ \kern3.25em AUC=V=P\left(c>i\right) $$

Substituting AUC for *V* in Eq. 4 produces a very simple formula for relating gamma and AUC:

8 $$ \kern4em Gamma=2 AUC-1 $$

Moreover, we can estimate AUC using Eq. 5 for *A*_*g*_, and then *A*_*g*_ can be substituted in Eq. 8 in order to obtain an estimate of gamma:

9 $$ {\displaystyle \begin{array}{c} Gamma=2\left[0.5\sum \limits_{k=0}^n\left(H{R}_{k+1}+H{R}_k\right)\left( FA{R}_{k+1}- FA{R}_k\right)\right]-1\\ {}=\left[\sum \limits_{k=0}^n\left(H{R}_{k+1}+H{R}_k\right)\left( FA{R}_{k+1}- FA{R}_k\right)\right]-1\end{array}} $$

Equation 9 provides an alternative method for computing gamma that is no more complex to compute than the original formula proposed by Goodman and Kruskal (1954), but that does not rely on the concepts of concordance and discordance. Consequently, it is not subject to the associated problem of ties. However, it is also well known that *A*_*g*_ has its own problems under certain circumstances (e.g., Grier, 1971; Simpson & Fitter, 1973). Because the trapezoidal rule necessitates drawing straight lines between the points on the ROC curve, AUC will be underestimated if the ROC is curvilinear, which is the usual case if the underlying evidence distributions are Gaussian. In short, the trapezoidal rule yields the minimum possible area under the ROC curve for a particular set of ROC coordinates. Some measures have been offered to compensate for this problem. For example, Donaldson and Good (1996) suggested *A’*_*r*_, which is the average of the minimum and maximum possible areas subtended by the ROC points. However, the computational procedure for this measure is considerably more complex than is that for *A*_*g*_, and it cannot be used for all data sets (e.g., there are slope restrictions). Consequently, for most of the remainder of this article, our aim is to compare the overestimation of true gamma caused by the concordance/discordance formula to the underestimation of true gamma caused by the trapezoidal rule, to determine which approach yields the better estimate. In the Discussion section, we will justify our nonparametric approach to this problem.

## Overview of the simulations

Our strategy for determining which measure provides the best estimate of gamma required us to compute each estimate for multiple simulated “participants” under a variety of circumstances and then to compare the results to a true measure of gamma. Henceforth, we refer to the estimate derived from concordant and discordant pairs as *G*_pairs_, the estimate based on ROC curves and the trapezoidal rule as *G*_trap_, and the true value of gamma as *G*_true_*. G*_pairs_ and *G*_trap_ were computed under conditions that simulated a variety of high-powered experiments, each with 100,000 participants and different parameter settings, as detailed later. To simulate realistic experimental conditions, each participant rated only 100 items (50 correct and 50 incorrect items; accuracy = 50%) drawn from Gaussian evidence distributions. The *SD* of the incorrect item distribution was fixed at 1.0 for all simulations, whereas the *SD* of the correct item distribution was varied. Confidence criteria were placed on the evidence dimension, and on each cycle of the simulation (corresponding to one participant), 50 items were randomly selected from each of the incorrect and correct evidence distributions and their subjective evidence values were evaluated with respect to the confidence criteria, to create a frequency table analogous to Table 1 or 2. The numbers of concordant and discordant pairs were computed from the data in the table, and *G*_pairs_ was computed using Eq. 1. To compute *G*_trap_, the data in the table were converted to cumulative frequencies, and the HRs and FARs at each confidence criterion were computed (Table 3). Once these rates had been obtained, *G*_trap_ was computed using Eq. 9. The end result was 100,000 estimates of both *G*_pairs_ and *G*_trap_, with each estimate being based on 100 items, from which the mean of each estimate could be computed for different underlying models with a varying set of parameters.

The next step was to compute *G*_true_ so that the accuracy of *G*_pairs_ and *G*_trap_ could be evaluated. There are a variety of methods to estimate *G*_true_. For the simplest (2 × 2) case, Masson and Rotello (2009) randomly selected 200,000 pairs of observations, one each from the correct and incorrect item distributions. They then compared the magnitudes of these two observations across all pairs, determining whether the pair was concordant or discordant (see Fig. 2), which allowed them to compute *G*_true_. Because real-valued numbers with high precision were used in these comparisons, there were few if any ties, thereby yielding an accurate gamma estimate.

Other methods can be used to estimate *G*_true_ that take advantage of the insights offered in this article regarding the relationship between AUC and *G*_true_. That is, *G*_true_ could be computed by first accurately estimating AUC and then converting that estimate to gamma by using Eq. 8. For example, if thousands of confidence criteria were used to derive *A*_*g*_, the process of computing the area becomes analogous to integration, so any underestimation of AUC would be negligible. However, an even better area estimate can be obtained by using the population parameters rather than by trying to minimize error in the sample estimate. Specifically, *A*_*z*_ can be computed if the ROC curve is transformed into a zROC by calculating *z*-scores corresponding to each HR and FAR pair plotted on the ROC. If the evidence distributions are Gaussian, as they were in all our simulations, the zROC becomes a straight line, intercepting both the *x*- and *y*-axes. If the slope and *y*-intercept of the population-based zROC are known, *A*_*z*_ can then be computed with the following equation (Stanislaw & Todorov, 1999; Swets & Pickett, 1982):

10 $$ {A}_z=\Phi \left[\frac{y\  intercept}{\sqrt{1+{(slope)}^2}}\right], $$

where Φ (“phi”) is the function that converts *z*-scores into probabilities. Because we fixed the *SD* of the incorrect item distribution at 1.0 in all simulations, the *y*-intercept was equal to the standardized distance between the means of the incorrect and correct item distributions, divided by the *SD* of the correct item distribution. The slope of the population-based zROC was equal to one divided by the *SD* of the correct item distribution. *G*_*true*_ was then calculated by substituting *A*_*z*_ for AUC in Eq. 8. Because the *y*-intercept and slope in Eq. 10 were population parameters for Gaussian distributions that we defined a priori, this method provides a perfect measure of AUC, and hence a perfect measure of gamma (*G*_true_).

As we noted earlier, we ran a variety of simulations testing different model parameters. The first set of 18 simulations assumed equal-variance Gaussian evidence distributions, whereas the second set of 18 simulations assumed unequal variances (total = 36 simulations). Specifically, the ratios of the *SD*s of the incorrect and correct item distributions in the first versus the second set of simulations were 1.0:1.0 and 1.0:1.25, respectively. An *SD* ratio of 0.8 (1.0:1.25) was chosen because research in recognition memory has demonstrated that a zROC with a slope of 0.8 fits the data well (e.g., Wixted, 2007).

Within each set of simulations, we varied three additional parameters: the number of points (confidence criteria) on the metacognitive scale, resolution, and bias. The number of scale points was either 6, 10, or 101, corresponding to commonly used 1–6, 1–10 (e.g., percentage scale on which only values evenly divisible by ten are permitted: 0%, 10%, 20%, . . . , 90%; Table 1), and 0–100 confidence scales, respectively.

Resolution was tested under two conditions, low and high, corresponding to standardized distances between the means of the evidence distribution of 0.5 and 2.0, respectively. In all simulations, the mean of the incorrect item distribution was fixed at 0 (*SD* = 1) on the evidence dimension. Thus, the means of the correct item distributions were 0.5 and 2.0 for the low- and high-resolution models, respectively.

Three levels of bias were tested: liberal, unbiased, and conservative. These different bias levels were created by varying the placement of the confidence criteria on the evidence dimension. To determine the placements, we first specified the locations of the highest and lowest criteria. The lowest, most liberal criterion for any dataset necessarily yields an HR–FAR pair corresponding to the (1,1) point on the ROC (see Figs. 1 and 2 and the bottom panel of Table 3). This occurs because confidence judgments are usually required for all items, which means that 100% of both incorrect and correct items are assigned the lowest level of confidence or higher. Because the HR and FAR are necessarily equal to 1.0 regardless of the model assumed, it was not informative to include this criterion in the simulations. Instead, the lowest criterion was associated with the second lowest value on each scale. This criterion was placed at – 2.0 on the evidence dimension for the liberal and unbiased cases, and at 0.0 for the conservative case (i.e., at the mean of the incorrect item distribution). The highest criterion for the unbiased and conservative cases was equal to the resolution value (either 0.5 or 2) plus two times the *SD* of the correct item distribution. For the liberal case, the highest criterion was equal to the resolution value (i.e., at the mean of the correct item distribution). The remaining criteria, the number of which varied according to which type of scale was being simulated, were spaced at equal intervals between the highest and lowest criteria. This methodology ensured that criteria were spread across the full range of both distributions if responding was unbiased, regardless of resolution or the *SD* of the correct item distribution. It also ensured that both the lowest HR for the liberal case and the highest FAR for the conservative case were equal to 0.5, again, regardless of the other parameters that were varied.

**Fig. 3:**
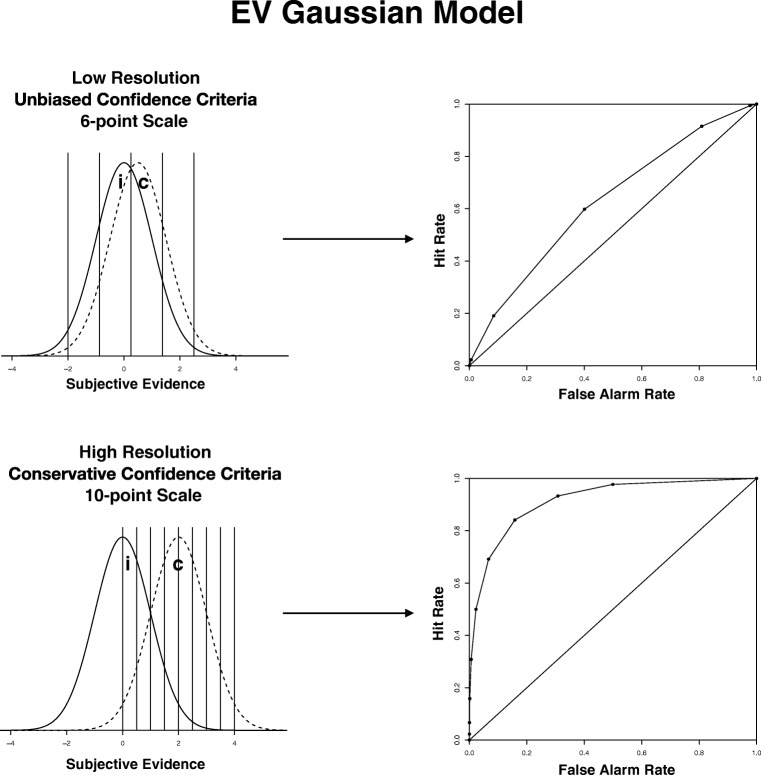
Graphical depictions of two of our simulations assuming equal-variance Gaussian evidence distributions. In each panel, the left side depicts the evidence distributions with confidence criteria, whereas the right side shows the associated ROC curve. The top panel shows the distributions with five criteria (6-point scale) that are unbiased, and resolution is low (standardized difference between means = 0.5). The bottom panel shows the simulation with nine criteria (10-point scale) that are conservative and where resolution is high (standardized difference between means = 2). EV = equal-variance; *c* = correct item distribution; *i* = incorrect item distribution

Schematic depictions of several models with different parameters and their associated ROC curves are shown in Figs. 3 (equal-variance model) and 4 (unequal-variance model). The top panel of Fig. 3 displays the equal-variance model corresponding to unbiased responding, a 6-point scale, and low resolution. The bottom panel displays the equal-variance model corresponding to conservative responding, high resolution, and a 10-point scale. In comparing the bottom panel with the top panel, note that the ROC curve is considerably more bowed in the bottom panel, which occurred because of the higher level of resolution. Also, the confidence criteria are shifted to the right (most liberal criterion at 0 rather than – 2 on the evidence dimension). This means that the points on the ROC do not represent the full range over which the items are distributed on the underlying evidence dimension. However, at high levels of resolution, this incomplete representation does not appear to affect the ROC much. That is, even though the conservative responding means that the highest FAR is only 0.5 on the ROC, the high resolution means that the HR is already close to 1.0.

**Fig. 4:**
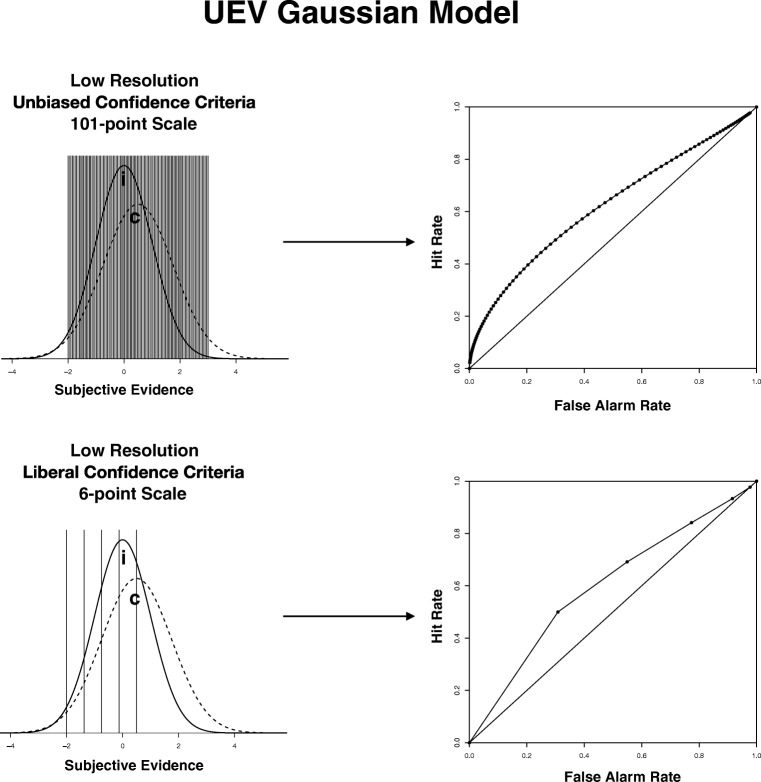
Graphical depictions of two of our simulations assuming unequal-variance Gaussian evidence distributions (1:1.25 ratio for *c* and *i* standard deviations, respectively). In each panel, the left side depicts the evidence distributions with confidence criteria, whereas the right side shows the associated ROC curve. The top panel shows the distributions with 100 criteria (101-point scale) that are unbiased and where resolution is low (standardized difference between means = 0.5). The bottom panel shows the simulation with five criteria (6-point scale) that are liberal and where resolution is low (standardized difference = 0.5). UEV = unequal-variance; *c* = correct item distribution; *i* = incorrect item distribution

Now consider the schematic depictions of the unequal-variance model shown in Fig. 4. The top panel corresponds to the case of a 101-point scale, low resolution, and unbiased responding. Note that the ROC for the unequal-variance case is not symmetric with respect to the chance diagonal, unlike the ROCs associated for the equal-variance models in Fig. 3. Note also that with a 101-point scale, the distances between the points on the ROC are much smaller, which should yield an accurate estimate of *G*_trap_ because very little of the true AUC is cut off by the straight lines joining the ROC coordinates. In contrast, the model in the bottom panel has a similar level of low resolution, but there are only five criteria (corresponding to a 6-point scale) and responding is liberal. Comparing the bottom panel with the top one, note that the large distance between the points on the ROC coupled with the liberal responding means that very few points represent the ROC in the conservative (bottom-left) region, where the bowing is greatest. Consequently, the straight line joining the most conservative ROC point and the (0,0) point cuts out a significant amount of area, suggesting that *G*_trap_ may not be very accurate in cases of low resolution, few confidence criteria, and liberal responding. We will return to this point later.

## Results

### Equal-variance model

**Fig. 5:**
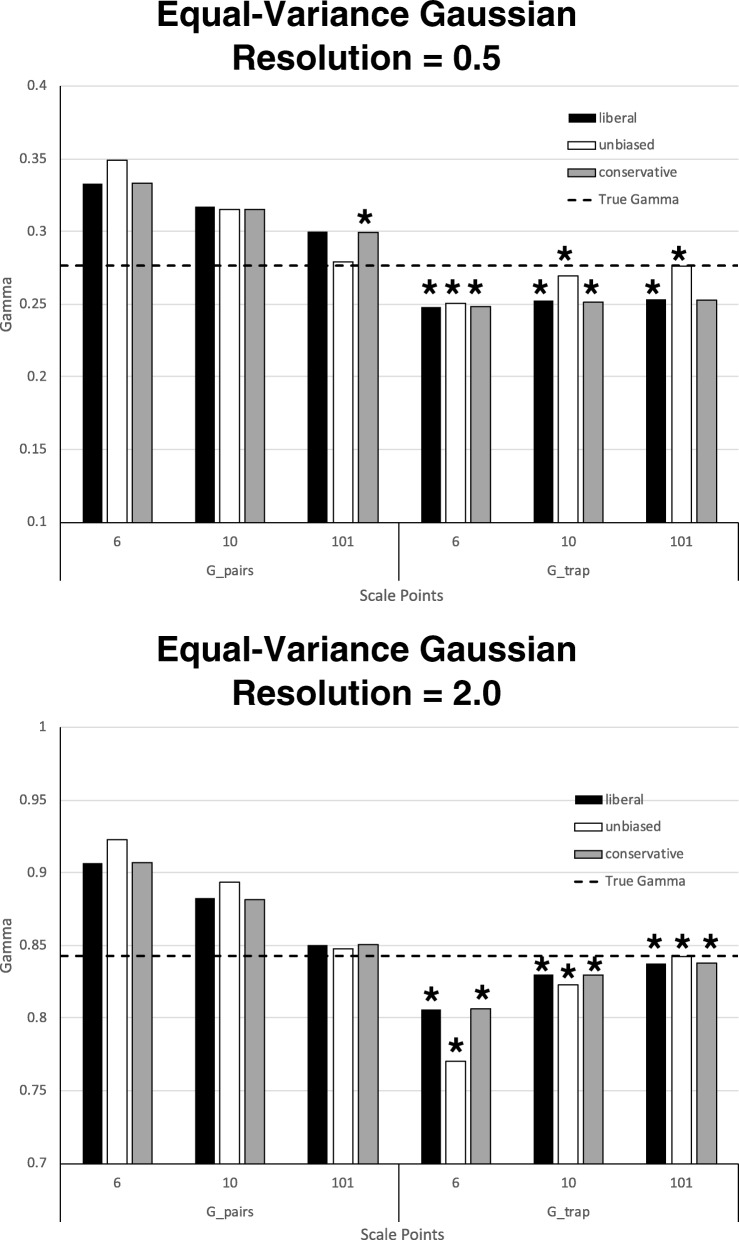
Means for two gamma estimates across 18 simulations (each based on 100,000 virtual participants) assuming equal-variance Gaussian evidence distributions. Low resolution (standardized difference between means of the signal and noise distributions = 0.5) is shown in the top panel, whereas high resolution (standardized difference = 2.0) is shown in the bottom panel. At each level of resolution, response bias and the number of scale points were varied. The true value of gamma is the horizontal dashed line in each panel. Asterisks indicate which of the two gamma estimates (*G_trap* = gamma estimated via ROC curves and the trapezoidal rule, *G_pairs* = gamma estimated by the original concordance/discordance formula) deviated the least from true gamma

The results of the simulations for the equal-variance model are shown in Fig. 5. The top versus bottom panels of Fig. 5 display the results for low (0.5) versus high (2.0) resolution, respectively. *G*_true_ is shown as the horizontal dashed line in each panel. Note that in all cases, regardless of the resolution level, *G*_pairs_ overestimated *G*_true_, whereas *G*_trap_ underestimated it. Note also that as the number of points on the scale increased, the accuracy of both estimates improved (i.e., the unsigned deviation from *G*_true_ was reduced). Unsurprisingly, increasing resolution had the effect of substantially increasing both *G*_true_ and the two estimates of gamma.

On the other hand, the effect of bias on each estimate was less straightforward. First consider the effect of bias at low resolution (top panel of Fig. 5). For *G*_pairs_, unbiased responding led to *poorer* estimates than did conservative or liberal responding for the 6-point scale, *equivalent* estimates for the 10-point scale, and *better* estimates for the 101-point scale. On the other hand, for *G*_trap_, unbiased responding led to better estimates than either conservative or liberal responding regardless of the number of scale points. However, this advantage for unbiased responding increased as the number of scale points increased.

Now consider the effect of bias at high resolution (bottom panel of Fig. 5). For *G*_pairs_, the pattern was similar to the pattern observed at low resolution. That is, unbiased responding led to worse estimates than either liberal or conservative responding for the 6-point scale. This difference was reduced for the 10-point scale and was slightly reversed for the 101-point scale, although all estimates with 101 scale points were close to *G*_true_. For *G*_trap_, the pattern was opposite to that observed at low resolution. That is, unbiased responding produced worse accuracy than either conservative or liberal responding for the 6-point scale, the difference was reduced for the 10-point scale, and slightly reversed for the 101-point scale. However, as with *G*_pairs_, all levels of bias produced estimates that deviated little from *G*_true_ for scales with a large number of response categories.

Most important for the present purposes is the *relative* accuracy of *G*_trap_ and *G*_pairs_. To facilitate this comparison, asterisks have been added above the data points in both panels of Fig. 5 to indicate which estimate produced the least unsigned deviation from *G*_true_. As Fig. 5 shows, *G*_trap_ yielded a better estimate in eight out of nine cases for low resolution (89%) and in nine out of nine cases for high resolution (100%; total for the equal-variance model = 17/18 = 94%).

### Unequal-variance model

**Fig. 6:**
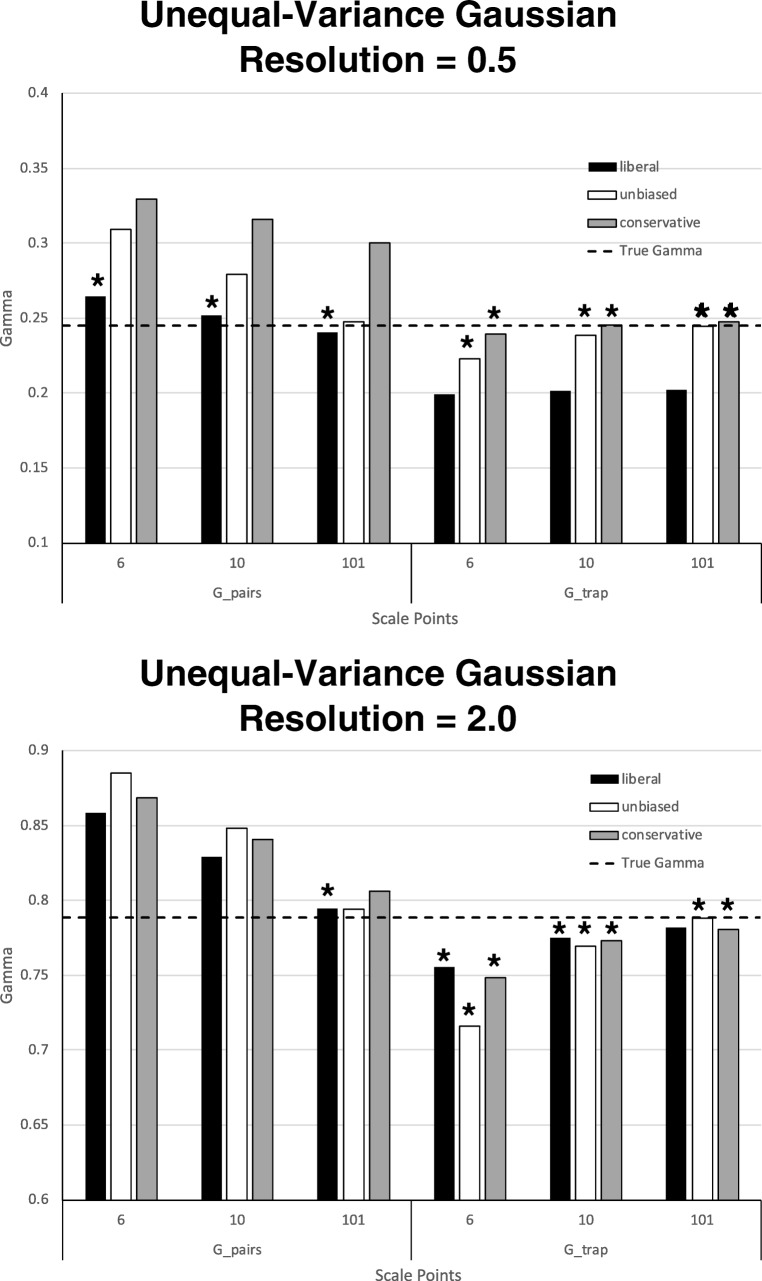
Means for two gamma estimates across 18 simulations (each based on 100,000 virtual participants) assuming unequal-variance Gaussian evidence distributions (1:1.25 ratio for signal and noise standard deviations). Low resolution (standardized difference between means of the signal and noise distributions = 0.5) is shown in the top panel, whereas high resolution (standardized difference = 2.0) is shown in the bottom panel. At each level of resolution, response bias and the number of scale points were varied. The true value of gamma is the horizontal dashed line in each panel. Asterisks indicate which of the two gamma estimates (*G_trap* = gamma estimated via ROC curves and the trapezoidal rule, *G_pairs* = gamma estimated by the original concordance/discordance formula) deviated the least from true gamma

The results of the simulations for the unequal-variance model are shown in Fig. 6. As with Fig. 5, the top versus bottom panels of Fig. 6 show the results for low (0.5) versus high (2.0) resolution, respectively, and *G*_true_ is shown as the horizontal dashed line in each panel. As with the equal-variance model, *G*_pairs_ tended to overestimate *G*_true_, whereas *G*_trap_ tended to underestimate it. Also as before, increasing resolution increased *G*_true_ and both gamma estimates. Generally speaking, increasing the number of points on the scale improved both gamma estimates, which also was true of the equal-variance model.

The effect of bias was again less straightforward. For *G*_pairs_ at low resolution, liberal responding tended to give the best estimates, with the exception of the 101-point scale condition, for which unbiased responding was best. The same pattern was evident for high resolution. For *G*_trap_ at low resolution, on the other hand, conservative responding tended to produce the best estimates, with the exception of the 101-point scale, for which unbiased responding was slightly better. However, at high resolution, liberal and conservative responding produced approximately equal levels of *G*_trap_ accuracy, regardless of the type of scale. Compared to biased responding, unbiased responding produced worse *G*_trap_ accuracy if the number of points on the scale was low (e.g., 6-point scale), but slightly better accuracy if the number of points on the scale was high (101-point scale).

Asterisks are again displayed in Fig. 6 to indicate which of the two gamma estimates, *G*_pairs_ or *G*_trap_, was more accurate (i.e., produced the lesser unsigned deviation from *G*_true_). For low resolution, *G*_trap_ was more accurate than *G*_pairs_ in six out of nine cases (67%). The exceptions were cases of liberal responding. The reason that liberal responding produced poor estimates of *G*_trap_ with the unequal-variance model at low resolution can be understood by examining the bottom panel of Fig. 4. With an unequal-variance model, the ROC bows more from the diagonal in the conservative region (i.e., the region associated with low HR and FAR values) than in the liberal region (i.e., the region associated with high HR and FAR values). However, because responding is liberal, there are few (or no) points on the ROC representing that bowed region. Consequently, the straight line extending from the most conservative ROC point to the (0,0) point cuts out a significant portion of the most bowed region of the ROC, causing *G*_trap_ to underestimate *G*_true_.

For high resolution, *G*_trap_ was more accurate than *G*_pairs_ in eight out of nine cases (89%). The exception was again a case of liberal responding in which, as with low resolution, there were few (or no) points representing the conservative region of the curve. However, as we noted earlier, the impact of this poor representation in the high-resolution case was not as great as in the low-resolution case, due to the nature of the ROC curves (i.e., the magnitude of the reversal was very small: 0.0008). The intuition for this fact can be obtained by examining the bottom panel of Fig. 3.^2^ Although there are no points representing any part of the subjective evidence dimension lower than 0 (where FAR = 0.5), the impact on *G*_trap_ is small because almost the whole of the correct item distribution has been mapped out at higher evidence levels. In other words, the HR is close to 1.0 at the most liberal confidence criterion, even though all the confidence criteria are quite far up the subjective evidence dimension.

## Discussion

There has been decades-long debate between the so-called *probabilistic* and *signal detection* camps regarding the best measure of metacognitive monitoring. The former camp, mostly led by Nelson (1984, 1986, 1987), has promoted gamma computed with Goodman and Kruskal’s (1954) original concordance/discordance formula. As an alternative, others have suggested using area or distance measures derived from SDT (e.g., Benjamin & Diaz, 2008; Higham, 2007, 2011; Masson & Rotello, 2009; Swets, 1986). We have provided mathematical proof that the two approaches are far more similar than has previously been assumed (see the supplementary materials). Specifically, true gamma is simply a linear function of the true area under the ROC curve (see Eq. 8). This means that both gamma and AUC in their true form are sensitive to the same metacognitive information and correlate perfectly across both participants and items. Thus, in their true form, there is no logical basis for preferring one measure over the other.

If the two measures are essentially the same, why have their relative merits been a subject of contention in the literature for so long? The problem lies not with the inherent superiority of one approach over the other. Instead, the problem lies in the *method used to estimate the true values*. Under the probabilistic approach, gamma has traditionally been estimated using the concepts of concordant and discordant pairs. Conversely, signal detection measures have typically been derived by estimating the distance between the signal and noise distributions (e.g., *d'* or *d*_*a*_) or AUC (e.g., *A*_*z*_ or *A*_*g*_). All of these measures are imperfect to varying degrees. The original gamma formula is distorted by ties and can overestimate the true gamma value quite substantially, particularly if there are only a few points on the metacognitive scale. *A*_*g*_ underestimates the true area under the ROC curve, particularly if there are few scale points and resolution is high. *A*_*z*_ and *d*_*a*_ provide accurate measures of discriminability as long as the underlying distributions are normal. However, if the normality assumption is violated, these measures also become grossly inaccurate. Hence, the question that researchers must ask themselves is not whether they should compute gamma versus some signal detection measure of resolution, as if these are opposing alternatives. The question should be which method should be used to estimate the true value of gamma, distance, or AUC in a given research context.

In an attempt to address this important question, we conducted 36 simulations involving 3,600,000 virtual participants to compare the relative accuracy of gamma computed with the original concordance/discordance formula against gamma computed with ROC curves and the trapezoidal rule. In all but five of these simulations, the method of computing gamma using area under the ROC curve was superior. That is, compared to gamma estimated with the concordance/discordance formula, computing AUC with the trapezoidal rule, doubling it, and subtracting one yielded less unsigned deviation from the true gamma value in 86% of our simulations. This superiority was true for myriad conditions. Across the 36 simulations, we manipulated the relative variances of the correct and incorrect item distributions, response bias, resolution, and the number of response categories on the confidence scale. The fact that ROC curves yielded the better gamma estimate across all these different conditions suggests that gamma computed in this way can be considered, in general, to be a better estimate of resolution than gamma computed with the original formula. Consequently, the former should be favored as the method of estimating resolution except in very specific circumstances (see the Limitations section).

Although the difference in the amounts that *G*_pairs_ and *G*_trap_ deviated from *G*_true_ may seem negligible in some cases, particularly if a large number of scale values were used, the *relative* deviations were not. To illustrate, we compared the unsigned deviations (from *G*_true_) for *G*_trap_ and *G*_pairs_ for the 31 (of 36) cases in which *G*_trap_ had higher accuracy. These comparisons indicated that *G*_trap_ was 3.41, 20.54, 34.56, and 4.06 times more accurate than *G*_pairs_ in the equal-variance/low-resolution, equal-variance/high-resolution, unequal-variance/low-resolution, and unequal-variance/high-resolution simulations, respectively.

Other criticisms might be that researchers, for the most part, are interested in whether gamma differs between experimental conditions or whether it is significantly different from zero, not in the true value of gamma. Given these interests, why is it so important to be concerned about accurate measurement of gamma? Our response to the first criticism is that the over/underestimation of gamma is not consistent across different contexts, which could result in spurious experimental differences being reported. As our opening example in the introduction reveals, *G*_pairs_ is generally greater for smaller than for larger contingency tables, *even for the same data set*. Thus, if gamma computed in an experimental condition with data arranged in a small contingency table (e.g., Report/Withhold × Accurate/Inaccurate) is compared to gamma in another experimental condition with data arranged in a larger contingency table (e.g., 1–6 Confidence × Accurate/Inaccurate), the former is likely to be larger than the latter purely as an artifact of the table size. Regarding the second criticism, overestimation or underestimation of gamma could produce spurious differences when gamma is compared against zero, leading researchers to conclude that gamma is above or below chance, respectively, when in fact it is not. This problem is particularly evident with small contingency tables. In our view, for these reasons and others, it is always preferable to estimate gamma as accurately as possible.

The number of points on the metacognitive scale was one of the most important factors affecting the accuracy of both *G*_pairs_ and *G*_trap_. Nelson (1984) argued that, although a correction may be needed for 2 × 2 tables so that the sample gamma (*G*_pairs_) is an unbiased estimate of the population gamma (*G*_true_), corrections were not needed for larger tables. The simulations reported here indicate that this statement is clearly not true; a 2 × 6 table, associated with a 6-point scale, showed large overestimations for *G*_pairs_. There was also a moderate amount of overestimation for the 10-point scale (10 × 2 table). Even the 101-point scale (101 × 2 table) yielded a small amount of overestimation, particularly if there was response bias. *G*_trap_ fared somewhat better but was also most distorted with the fewest scale points.

How might researchers overcome the estimation problem associated with few values on a metacognitive scale? One obvious option would be to ensure that experimental participants are provided with a full percentage scale and are encouraged to use any value between 0 and 100. Our simulations showed that these scales led to accurate estimates. One potential drawback with this approach is the introduction of measurement error: Scales with many values tend to have lower reliability than those with fewer points (e.g., Bishop & Herron, 2015). Another issue is that people tend to prefer 10-point scales (e.g., Preston & Colman, 2000). Therefore, given the opportunity, 101-point scales may be reduced to 10-point scales (i.e., participants only respond with values that are evenly divisible by 10: 10, 20, 30, etc.). To avoid these issues, an alternative approach may be to avoid explicit response categories altogether by having participants use a graphical interface to make metacognitive ratings. For example, if a computer is used to collect metacognitive ratings such as JOLs in an experimental setting, participants may be presented with a “slider” on the computer screen with labels ranging from *not at all likely to remember* on the far left to *very likely to remember* on the far right (see, e.g., Metcalfe & Miele, 2014). The number of pixels between the starting point at the far left of the scale to the point at which participants click to indicate confidence could then be calculated as a confidence measure. With modern computers, this would amount to a scale with even more points than a scale with 101 response categories and might avoid excessive measurement error and participants’ tendency to simplify scales with a large number of explicit numerical values.

### Variability of measures

Nelson (1984) argued that *A*_*g*_ is too variable to be used in most metacognitive experiments because of the limited number of items. In Nelson’s own words: “for nonparametric SDT to be appropriate in the feeling-of-knowing situation, it will be necessary to have many more observations per subject than currently are obtained” (pp. 122–123). Later, he argues that in most metacognitive experiments “the typical number of observations has been roughly one or two dozen per subject. . . . This number of observations, particularly when divided up via multilevel feeling-of-knowing ratings, is much too small for nonparametric SDT” (p. 123).

Thus, according to Nelson (1984), it is not possible to obtain a stable per-participant estimate of resolution unless there are 100 or more observations, due the inherent variability of *A*_*g*_ (and hence *G*_trap_). However, in our view, the more appropriate approach to understanding the effect of variability would be to *compare* the relative variability of measures such as *G*_pairs_ and *G*_trap_ rather than focusing solely on one measure or the other. Our simulations allowed us to do just that; that is, it was possible to compare the between-subjects standard deviations for both *G*_pairs_ and *G*_trap_ across our 100,000 virtual participants in each simulation. The results of this comparison indicated that, for both the equal- and unequal-variance Gaussian models with low resolution (standardized distance between the evidence distributions = 0.5), there was *less* variability for *G*_trap_ than for *G*_pairs_ in all cases, whereas the opposite was true for all cases of high resolution (standardized distance = 2.0). However, if the magnitudes of the differences are considered, *G*_trap_ was the less variable measure overall; that is, collapsing over the equal- and unequal-variance models, the mean advantage that *G*_trap_ had over *G*_pairs_ at low resolution was 0.023, whereas the mean advantage that *G*_pairs_ had over *G*_trap_ at high resolution was only 0.008, nearly a threefold difference.

One criticism with this analysis is that each of our simulations involved 100 items (50 correct, 50 incorrect), and Nelson (1984) claimed that 100 items or more would make nonparametric SDT analyses acceptable. Hence, the real question is how the variability of each gamma estimate compares when there are fewer items. To answer this question, we repeated all 36 simulations reported earlier with only 20 items per participant (10 correct, 10 incorrect). We also reduced the number of virtual participants from 100,000 per simulation to just 40. If Nelson’s claims are correct, then the variability of *G*_trap_ should become large and unmanageable with these parameter settings and should far exceed that of *G*_pairs_. However, although the per-participant standard deviations increased with the reduction in items, they increased for both *G*_trap_ and *G*_pairs_. In terms of the comparison of the two measures, the results were very similar to the previous results; that is, there was *less* variability for *G*_trap_ than for *G*_pairs_ for both the equal- and unequal-variance Gaussian models in all cases at low resolution, whereas the opposite was true for all cases of high resolution. Again, however, if the magnitudes of the differences are considered, *G*_trap_ was the less variable measure overall. As before, collapsing over the equal- and unequal-variance models, the mean advantage that *G*_trap_ had over *G*_pairs_ at low resolution was 0.020, whereas the mean advantage that *G*_pairs_ had over *G*_trap_ at high resolution was 0.018.

Overall, these comparisons of the between-subjects standard deviations of *G*_trap_ and *G*_pairs_ indicate that, if anything, *G*_trap_ is the less variable measure regardless of the number of items or the number of virtual participants that contribute to the estimates, at least with Gaussian evidence distributions. Hence, there is no evidence that nonparametric SDT should be rejected on the basis of high variability, as Nelson (1984) claimed, regardless of whether one is computing *A*_*g*_ or *G*_trap_ as the measure of resolution.

### Parametric versus nonparametric measures of resolution

As we noted earlier, if the underlying evidence distributions are Gaussian and the true (population) values of the zROC’s *y*-intercept and slope are entered into Eq. 10, *A*_*z*_ is a *perfect* estimate of AUC. Indeed, the *A*_*z*_ value from Eq. 10 was substituted for AUC in Eq. 8 in order to compute *G*_true_ for our simulations, the gold standard against which *G*_trap_ and *G*_pairs_ were compared. Why, then, did we use the trapezoidal rule to estimate gamma in our simulations rather than *A*_*z*_, particularly since we assumed Gaussian distributions for our simulations, anyway? There were two reasons for this decision. First, very little is known about the nature of the evidence distributions in metacognition. In one of the few formal tests that have been conducted to determine the nature of these distributions, Higham (2007) found that an equal-variance Gaussian model was a good fit for the metacognitive ROC curves generated by performance on the SAT. However, whether this finding is generally true across the myriad ratings that are used in modern metacognitive research is an open question.

Furthermore, some authors have suggested that signal detection measures of resolution are inappropriate in the first place, because there may be only a single distribution of items rather than two (signal and noise). The reasoning here seems to be that, unlike in tasks that lend themselves easily to signal detection analyses, such as old–new recognition, there are no distractors in the usual sense of the word in recall tasks; therefore, there is only one distribution of items (e.g., Murayama, Sakaki, Yan, & Smith, 2014, note 1). The spirit of this single-distribution assumption is captured in Jang, Wallsten, and Huber’s (2012) stochastic model of JOL accuracy. However, in our view, this reasoning confuses Type 1 (stimulus-contingent) and Type 2 (response-contingent) discrimination. Metacognitive discrimination is essentially a Type 2 SDT task involving accuracy discrimination, so distractors are not defined by their stimulus characteristics (e.g., old vs. new items), but rather by their response characteristics (e.g., correct vs. incorrect responses on a criterial test). In the context of recall, then, the distractors are errors of commission or omission on the memory test (see Arnold et al., 2013; Higham, 2007, 2011, for discussion).

Nonetheless, for the present purposes, the important point is that there is some doubt regarding the nature of the evidence distributions. Consequently, we thought it would be hasty to jump to the conclusion that the distributions are unquestionably Gaussian. Such an assumption seems *plausible*, which is why we adopted it for the simulations that we reported, but it is not a *certainty*.^3^ Because neither *G*_trap_ nor *G*_pairs_ is reliant on any particular evidence distribution shape, Gaussian or otherwise, these were the measures we chose to compare. However, it should be noted that if the ROC data conform to a Gaussian model—and there are fairly straightforward statistical methods for testing this assumption (see, e.g., DeCarlo, 2003)—then gamma estimated via *A*_z_ would certainly be more accurate than gamma estimated via *A*_*g*_.

The second reason we focused on nonparametric measures is more pragmatic. Unlike recognition tasks, in which the number of targets and distractors making up the signal and noise distributions are defined a priori by the experimenter and are often equated (i.e., 50% targets, 50% distractors), the correct versus incorrect evidence distributions in metacognitive applications of SDT are determined by participants’ accuracy on the criterial test. Depending on the experimental circumstances, accuracy can be extreme, which would result in only a few items populating one distribution or the other. The high variability in HRs and FARs derived from only a few items in cases of extreme accuracy can result in many zeroes and/or ones in the dataset. For example, suppose participants are engaged in a very difficult recall task with 100 items and they are informed in advance that the test will be difficult. Because the memory test is hard, suppose that accuracy is only 10%. Furthermore, because participants are told about the difficulty of the upcoming memory test, the few correct responses that are made on the test are assigned the lowest JOL. Under these circumstances, all the HRs on the metacognitive ROC (apart from the [0,0] point) would be equal to 10/10 = 1.0.

The problem with HRs and/or FARs equal to either 0 or 1 is that parametric estimates such as *d'*, *d*_*a*_, and *A*_*z*_ are undefined. Of course, some commonly used corrections can be applied to the frequencies prior to computing the HRs and FARs, to avoid 0s and 1s. However, when the frequencies underlying these rates are low, these corrections can distort the rates considerably (see Hautus, 1995, for cases of distortion caused by common corrections even when frequencies are not low). To illustrate, consider again the participant who produced only ten correct responses on a difficult recall test that were all assigned the lowest JOL. If the common 1/(2*N*) rule is applied, the HRs = 10/10 = 1.0 are corrected to 1.0 – 1/(2*10) = .95. If the participant’s performance was even worse, such that there were only five correct responses (5% recall accuracy), the 1/2(*N*) rule would adjust the HRs from 1.0 to .90. Although these examples are extreme (i.e., very few correct responses), they illustrate the point that in the context of metacognitive discrimination, the magnitude of the correction using the 1/2(*N*) rule is confounded with accuracy on the criterial test. Such confounding means that the correction would greatly distort all parametric indices if accuracy were extremely high or low. The situation would be even worse if *both* the HRs and the FARs required correction (as in cases of HR = 1.0 and FAR = 0). Critically, however, HRs and/or FARs equal to 0 or 1 do not need to be corrected at all in order to compute either *A*_*g*_ or *G*_trap_. For this reason, we recommend avoiding corrections altogether in the context of metacognitive research and relying on nonparametric estimates of resolution.

### Negative resolution

We have focused solely on positive relationships between metacognitive ratings and accuracy. However, in rare circumstances this relationship can be negative, such as when deceptive general-knowledge questions are used (e.g., Higham & Gerrard, 2005; Koriat, 2018). With such questions, people typically respond with, and are more confident in, incorrect rather than correct answers (e.g., many people confidently, but erroneously, believe that Sydney is the capital of Australia). This results in negative resolution, and if gamma is computed with the original concordance/discordance formula, it assumes values less than 0. Is computing gamma with ROC curves still possible under these circumstances? The short answer is “yes.” The ROC curves would bow below, rather than above the chance diagonal, yielding area measures that were less than 0.5. *G*_trap_ can be computed in the same way as before: doubling *A*_*g*_ and subtracting 1, resulting in negative *G*_trap_ values. To illustrate with an example, suppose that participants answer some deceptive questions and provide retrospective confidence ratings regarding the accuracy of their answers.^4^ Because they assign higher confidence ratings to incorrect than to correct responses, suppose that the area under the metacognitive ROC curve is only 0.3. If this value is doubled and 1 is subtracted from the product, the resultant gamma value would be 0.3*2 – 1 = – 0.4. In the extreme case, AUC would be equal to 0 and gamma would be equal to – 1.

### Limitations

One drawback to computing *G*_trap_ instead of *G*_pairs_ is that *G*_trap_ can only be used in situations in which there are two outcomes on the criterial test (e.g., correct vs. incorrect recall). Hence, *G*_trap_ cannot be used to estimate resolution for criterial tests such as trials to criterion or reaction times. However, the vast majority of research in metacognition focuses on resolution computed with respect to correct and incorrect responses, so this is unlikely to pose a significant problem in most situations.

Our simulations showed that *G*_trap_ does not perform well if there is a combination of low resolution, unequal-variance Gaussian evidence distributions, and liberal responding. With this combination of factors, *G*_trap_ is a poorer estimate of *G*_true_ than is *G*_pairs_. Indeed, four of the five cases in which *G*_trap_ was less accurate than *G*_pairs_ in our simulations occurred with the unequal-variance Gaussian model and liberal responding. The best way to identify cases such as these is to construct an ROC curve of the data, as such curves provide information pertaining to the levels of all three variables: Resolution is indicated by the extent to which the ROC curve bows from the chance diagonal; the shape of the ROC curve gives an indication of the nature of the underlying evidence distributions (and can be formally evaluated using a goodness-of-fit test); and the level of bias can be determined by where the points are clustered on the ROC. Of course, there are limitations to this analysis, as well. For example, if responding is highly biased, portions of the ROC curve will not be represented by any points, so it will be difficult or impossible to get an accurate indication of the full shape of the ROC curve. Nonetheless, if the ROC coordinates are clustered in either the bottom left (conservative) or top left (liberal) portion of the ROC, then researchers will be alerted to response bias. More generally, ROC curves usually provide an excellent visual representation of metacognitive data. In our view, constructing an ROC should be the first step researchers take when deciding on an analysis strategy.

#### Author note

Portions of this research were presented at the 56th Annual Meeting of the Psychonomic Society, Chicago, IL.

## Electronic supplementary material

ESM 1 (PDF 145 kb)

## Footnotes

1 The evidence dimension in SDT is commonly described as “memory strength.” This has typically caused SDT to be rejected by metacognitive theorists because that label seems to imply that people make metacognitive judgments on the basis of direct access to the contents of memory (e.g., Koriat, 2012). However, as is discussed in Higham et al. (2016), there is no need to equate the underlying dimension with memory strength. It is better to consider the dimension as reflecting all sources of influence that participants subjectively consider relevant to correctness. These influences can be based on memory access or can be based on myriad other metacognitive cues that are more inferential in nature (e.g., font size; see Luna, Martín-Luengo, & Albuquerque, 2018).

2 Although Fig. 3 depicts an equal-variance model, it still highlights the point that conservative responding has little effect on *G*_trap_ if resolution is high.

3 Apart from this plausibility, the other reason that we assumed Gaussian distributions for our simulations was that this assumption would provide a conservative test of *G*_trap_ accuracy. It is well-known that the trapezoidal rule underestimates AUC if the ROC is bowed, as it is with Gaussian distributions. If *G*_trap_ performs well under these circumstances (which it did, at least relative to *G*_pairs_), it is likely that it would perform even better if there were a linear relationship between the HRs and FARs, which would occur if the underlying distributions were uniform.

4 We have focused mostly on JOLs throughout this article, but it is important to keep in mind that our arguments and simulation results apply equally to all types of metacognitive ratings, including retrospective confidence.
