# Supplementary material for: New improved gamma: Enhancing the accuracy of Goodman–Kruskal’s gamma using ROC curves
Source: Behav Res Methods. 2018 Sep 27;51(1):108–25. doi: 10.3758/s13428-018-1125-5 (PMC6420444; doi:10.3758/s13428-018-1125-5)
Supplement: Supplementary file 1 — (PDF 145 kb) [file 13428_2018_1125_MOESM1_ESM.pdf]

MATHEMATICAL EQUIVALENCY OF THE PROPORTION OF CONCORDANT PAIRS (V) AND THE AREA UNDER THE ROC CURVE

What follows is a proof that the proportion of concordant pairs in a sample (i.e., $P[c > i]$, where c and i represent randomly drawn observations from the correct and incorrect item distributions, respectively) is equal to the area under the ROC curve. The proof is general requiring only that the the distributions be Riemann integrable, hence not restricted to be Gaussian or even continuous. Nor do the distributions need to arise naturally as the signal and noise distributions as in signal detection theory. Therefore, we present the proof in very general terms.

Let X and Y be stochastically independent random variables with probability density functions f and g and distribution functions F and G respectively, so that

$$F(x) = \int_{-\infty}^{x} f(t)\,\mathrm{d}t \quad \text{and} \quad G(x) = \int_{-\infty}^{x} g(t)\,\mathrm{d}t$$

As a measure of how separated these probability distributions are we can sample X and Y and ask for the fraction of samples where $X < Y$, i.e., we want to find an expression for $\Pr(X < Y)$. This can be obtained by integrating the joint density function of X and Y over the region defined by $\{(x, y) : x < y\}$, which is illustrated in the following figure as the shaded region above the line $y = x$.

Since X and Y are stochastically independent, the joint density function of X and Y is the product of the marginal density functions and so has the value $f(x)g(y)$ at the point (x, y) in the Cartesian plane. Thus we have

$$\begin{aligned}\Pr(X < Y) &= \int_{-\infty}^{\infty}\int_{-\infty}^{y} f(x)g(y)\,\mathrm{d}x\,\mathrm{d}y \\ &= \int_{-\infty}^{\infty}\left[\int_{-\infty}^{y} f(x)\,\mathrm{d}x\right] g(y)\,\mathrm{d}y \\ &= \int_{-\infty}^{\infty} F(x)g(x)\,\mathrm{d}x \qquad (1)\end{aligned}$$

If there is a tendency for X to be less than Y, then it is reasonable to expect that the distribution function for X will tend to be greater than the distribution function for Y, since X will 'use up' its probability before Y does. It is natural then to consider the geometry of the points $\big(F(t), G(t)\big)$.

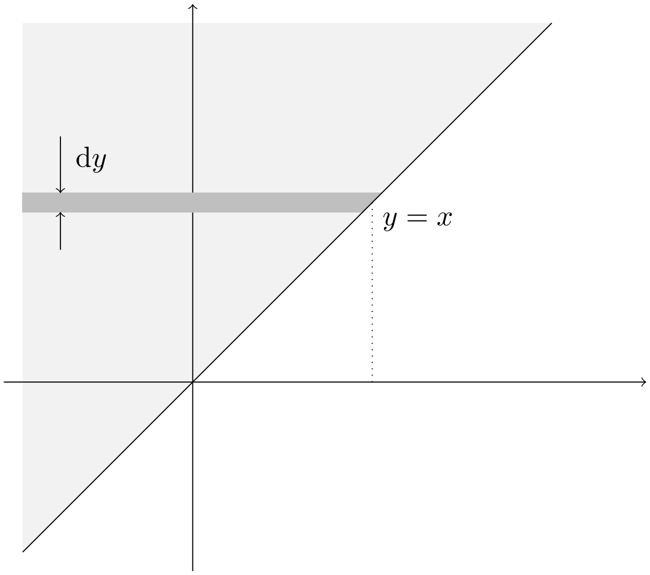

FIGURE 1. Region where $X < Y$

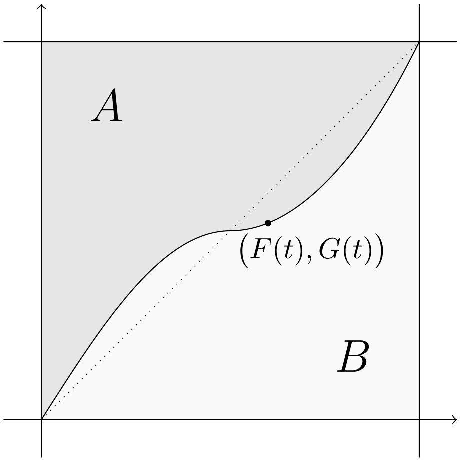

FIGURE 2. Unit square partition by the curve C

The set of points given by

$$C = \{\big(F(t), G(t)\big) : -\infty < t < \infty\}$$

is a curve joining the points $(0, 0)$ and $(1, 1)$ that always lies in the unit square, i.e., the region bounded by the x and y-axes and the lines $x = 1$ and $y = 1$. The curve C divides the unit square into the two regions A and B, above and below C.

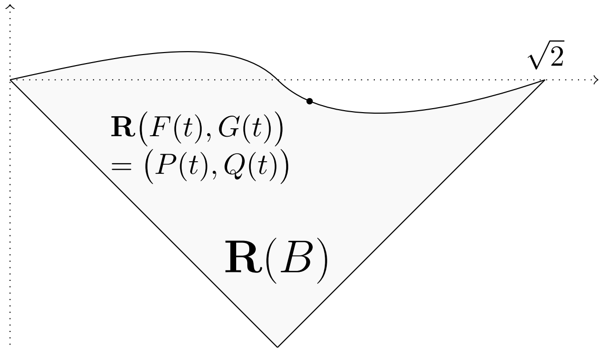

FIGURE 3. Rotated curve C

If $F(t) > G(t)$, then the point $\big(F(t), G(t)\big)$ lies below the main diagonal of the unit square, the line $y = x$. The greater the tendency for X to be less than Y, the more values of t will have $F(t) > G(t)$, the more time C will spend in this lower triangle and the greater will be the area of A.

To compute the area of A it will be convenient to use the fact that

$$area(A) = 1 - area(B) \tag{2}$$

and to compute $area(B)$ it will be convenient to rotate the plane first so that the main diagonal becomes horizontal, i.e., a clockwise rotation through $\frac{\pi}{4}$.

The desired rotation is an area-preserving linear transformation that is specified by the matrix

$$\mathbf{R} = \frac{1}{\sqrt{2}} \begin{bmatrix} 1 & 1 \\ -1 & 1 \end{bmatrix} \quad \text{and let} \quad \begin{bmatrix} P(t) \\ Q(t) \end{bmatrix} = \mathbf{R} \begin{bmatrix} F(t) \\ G(t) \end{bmatrix}$$

so that

$$\begin{aligned} P(t) &= \frac{1}{\sqrt{2}}\big(F(t) + G(t)\big) \\ Q(t) &= \frac{1}{\sqrt{2}}\big(G(t) - F(t)\big) \end{aligned}$$

Consider the area bounded by the curve $\mathbf{R}(C)$ and the (new) x-axis and call this area $A_{\mathbf{R}(C)}$. When the curve is above the x-axis it makes a positive contribution to the area and when it is below it makes a negative contribution. If we knew the function of x whose graph would be $\mathbf{R}(C)$, then the area computation would be a straightforward integral, but we only know the points on the curve in terms of t instead.

If the parameter makes a small displacement from t to $t + \Delta t$, we move along the curve from $\big(P(t), Q(t)\big)$ to $\big(P(t{+}\Delta t), Q(t{+}\Delta t)\big)$ and trace out a thin vertical slice of area between the curve and the x-axis. The area of this slice is approximately

$$Q(t)\big(P(t+\Delta t) - P(t)\big) = Q(t)\frac{P(t+\Delta t) - P(t)}{\Delta t}\Delta t$$

Now summing up all the thin slices and letting Δt approach 0, we have

$$A_{\mathbf{R}(C)} = \lim_{\Delta t \to 0} \sum Q(t)\frac{P(t+\Delta t) - P(t)}{\Delta t}\Delta t = \int_{-\infty}^{\infty} Q(t)P'(t)\,\mathrm{d}t$$

Substituting for P and Q and then rearranging terms gives

$$\begin{aligned}
A_{\mathbf{R}}(C) &= \int_{-\infty}^{\infty} Q(t)P'(t)\,\mathrm{d}t \\
&= \frac{1}{2}\int_{-\infty}^{\infty} \big(G(t) - F(t)\big)\big(G'(t) + F'(t)\big)\,\mathrm{d}t \\
&= \frac{1}{2}\int_{-\infty}^{\infty} \big[\big(G(t) + F(t)\big)\big(G'(t) + F'(t)\big) - 2F(t)\big(G'(t) + F'(t)\big)\big]\,\mathrm{d}t \\
&= \frac{1}{2}\int_{-\infty}^{\infty} \big(G(t) + F(t)\big)\big(G'(t) + F'(t)\big)\,\mathrm{d}t - \int_{-\infty}^{\infty} F(t)F'(t)\,\mathrm{d}t - \int_{-\infty}^{\infty} F(t)G'(t)\,\mathrm{d}t
\end{aligned}$$

Letting $u = G(t) + F(t)$ in the first integral and $v = F(t)$ in the second, we have

$$\begin{aligned}
&= \frac{1}{2}\int_{0}^{2} u\,\mathrm{d}u - \int_{0}^{1} v\,\mathrm{d}v - \int_{-\infty}^{\infty} F(t)g(t)\,\mathrm{d}t \\
&= 1 - \frac{1}{2} - \int_{-\infty}^{\infty} F(t)g(t)\,\mathrm{d}t \\
&= \frac{1}{2} - \int_{-\infty}^{\infty} F(x)g(x)\,\mathrm{d}x
\end{aligned}$$

Adding the area of the triangle below the x-axis, we have

(3) $$area(B) = area\big(\mathbf{R}(B)\big) = \frac{1}{2} + A_{\mathbf{R}(C)} = 1 - \int_{-\infty}^{\infty} F(x)g(x)\,\mathrm{d}x$$

Finally, combining equations (1), (2) and (3) we get

$$\boxed{\Pr(X < Y) = area(A)}$$

This result can now be applied to signal detection theory where the random variables X and Y represent the sensed intensity of noise and the sensed intensity of a mixture of signal and noise respectively. Instead of the distribution functions F and G, the quantities of interest are called the hit rate HR, and the false alarm rate FAR. These quantities are the complements of the respective distribution functions and we have that

$$\begin{aligned} x = \quad & FAR(t) & = 1 - F(t) \\ y = \quad & HR(t) & = 1 - G(t) \end{aligned}$$

are exactly the parametric equations for the ROC curve of X and Y.

Following the above equations, if the graphic depicted in Figure 2 is turned upside-down (reflected in the line $y = \frac{1}{2}$) and then the result flipped left-to-right (reflected in the line $x = \frac{1}{2}$), the final result will be the traditional depiction of the ROC curve as it usually sits in the unit square. Note that this final result will be the same as if the region had been rotated about the point $(\frac{1}{2}, \frac{1}{2})$, by π, i.e., a half revolution.

With this transformation the region A in Figure 2 starts above the curve C and ends up below the ROC curve and clearly has the same area. We conclude

$$\boxed{\Pr(X < Y) = AUC}$$
